# Treatment of glenohumeral internal rotation deficit in the general population with shoulder pain: An open single-arm clinical trial

**DOI:** 10.1097/MD.0000000000035263

**Published:** 2023-09-22

**Authors:** Rafael Jácome-López, Javier Tejada-Gallego, José María Silberberg, Fernando García-Sanz, Francisco García-Muro San José

**Affiliations:** a Physiotherapy Unit, Clínica Universidad de Navarra, Madrid, Spain; b CEU International Doctoral School (CEINDO), University San Pablo-CEU, Madrid, Spain; c Orthopaedic Surgery and Traumatology Department, Clínica Universidad de Navarra, Madrid, Spain; d Physiotherapy Service, Clínica CEMTRO, Madrid, Spain; e Department of Physiotherapy, Faculty of Medicine, University San Pablo-CEU, Boadilla del Monte, Spain.

**Keywords:** general population, glenohumeral internal rotation deficit, glenohumeral joint, patient outcome assessment, shoulder pain

## Abstract

**Background:**

Maladaptation can provoke important alterations in the arthrokinematics such as an internal rotation reduction in the dominant shoulder compared with the nondominant shoulder known as glenohumeral internal rotation deficit (GIRD). Though the number of studies investigating GIRD in athletic population, there are not studies reporting the efficacy of the GIRD treatment in the nonathlete population, a kind of study required to improve our understanding of patient care with this pathology. This study aimed to describe the efficacy of the GIRD treatment in nonathlete population with shoulder pain.

**Methods:**

An open single-arm trial with 35 patients was adopted for evaluating the efficacy of GIRD treatment in patients with shoulder pain. All patients with shoulder pain who attended the consultation, accepted, and agreed to participate in the study between October 2020 and March 2021 were included. A treatment sequence including joint manual therapy techniques and soft tissue release techniques was applied in the consultation. Then, patients were instructed to adapt the daily active biological stimulus at home. The IR before (IR_0_) and after (IR_1_) the treatment was considered the outcome measure. The GIRD was calculated as the difference between the IR of the non-painful shoulder and the IR of the painful shoulder before (GIRD_0_) and after treatment (GIRD_1_). A paired Student *t* test was used to compare the GIRD of each patient before and after the treatment.

**Results:**

Treatment of the patients significantly increased the IR of the painful shoulder in all the patients (*P*-value < .0001) So, the mean IR_0_ was 26.09 ± 14.46º (23.64–28.53), and after the treatment the mean IR_1_ was 67.98 ± 15.03º (65.48–70.52). The mean difference after the treatment (IR_1_–IR_0_) was 41.89 ± 14.74º (39.4–44.39). The treatment also significantly reduced GIRD (*P*-value < .0001). So, the mean GIRD_0_ was 42.95 ± 16.26º (40.2–45.7), and after the treatment the mean GIRD_1_ was –1.05 ± 17.18º (–3.96 to 1.85).

**Conclusions:**

The treatment administrated in this study significantly increased the internal rotation of the treated and painful shoulder and reduced the GIRD from the first consultation.

**Level of evidence:**

Level 3.

## 1. Introduction

Shoulder pain is a frequent disabling joint problem in the general population. The reported incidence ranges between 0.9% and 2.5%^[1]^ depending on age.^[2]^ Its point prevalence varies from 6.9% to 36%, however the life prevalence can be up to 66.7%.^[1]^ Almost a third of patients with shoulder pain need a sick leave,^[3]^ therefore, this pain is associated with a productivity loss derived from decreased performance at work.^[4]^

Shoulder pain is mainly related to the glenohumeral joint.^[5]^ Optimal glenohumeral rotation is crucial for glenohumeral arthrokinematics. This rotation is composed of both internal rotation (IR) and external rotation (ER). The appropriate balance of IR and ER maintains the humeral head centered in the glenoid fossa and increases concavity compression.^[6]^ Maladaptation can provoke important alterations in the arthrokinematics such as an IR reduction in the dominant shoulder compared with the nondominant shoulder known as glenohumeral internal rotation deficit (GIRD).^[6]^ The term GIRD is used to describe an IR measurement which is considered one of the most important factors related to injury risk in the arm of overhead athletes, such as baseball pitchers,^[7–9]^ tennis players,^[10]^ hammer throwers,^[11]^ shot putters,^[11]^ among others. Interestingly, some athletes with GIRD use to show pain in a higher frequency of the upper body, including shoulder pain.^[12]^ Recently, GIRD is showing a high incidence in patients from the general (nonathlete) population with shoulder pain (under review).

Recovery from this ailment is slow and is associated with a high recurrence or persistence pain at 12-month.^[13]^ Since GIRD is primarily a soft tissue problem, the aim of the treatment should ideally be focused on removing it. The treatment should include interventions related to muscle stiffness, inflexibility, muscle weakness, and capsular stiffness. Though multiple programs proposed to correct the rotational alterations demonstrated a good success,^[14–16]^ the sleeper stretch is the most used exercise to increase IR.^[17]^ The effectiveness of the stretches, mobilization techniques, and exercises depends on the athlete performing them daily throughout the playing season.^[18]^ This clearly demonstrated that the effectiveness of the treatment depends on the athlete’s perseverance and commitment to treatment. Kirsch et al^[19]^ pointed out a tendency related to the athletic population to prioritize studies investigating the epidemiology of GIRD and review articles above studies evaluating clinical, therapeutic, and patient-reported outcomes. Noteworthy that there is a complete lack of studies reporting the efficacy of the treatment of GIRD in the nonathlete population.

This study aimed to describe the efficacy of the GIRD treatment in nonathlete population with shoulder pain provoked by a soft tissue problem. Our hypothesis was that the treatment here described would be able to reduce considerably the GIRD in this kind of patients. Thus, to our knowledge, our work would be of the first studies to evaluate the treatment of GIRD in a nonathlete population, which would improve the therapeutic approach to patients with this pathology, as well as broaden the understanding of this condition for clinicians and researchers.

## 2. Materials and Methods

### 2.1. Study design

An open single-arm trial (clinical trial registration number: NCT05108311) was adopted for evaluating the efficacy of reducing GIRD in patients with shoulder pain using an exercise routine initiated at the time of the first consultation. All patients underwent a medical examination.

### 2.2. Ethics approval

The study was conducted in accordance with the Declaration of Helsinki and approved by the Ethics Committee of the Clínica Universitaria de Navarra (Project 2020.095 of June 18, 2020). Informed consent was obtained from all subjects and/or their legal guardian(s) for publication of identifying information/images in an online open-access publication.

**Figure 1.**
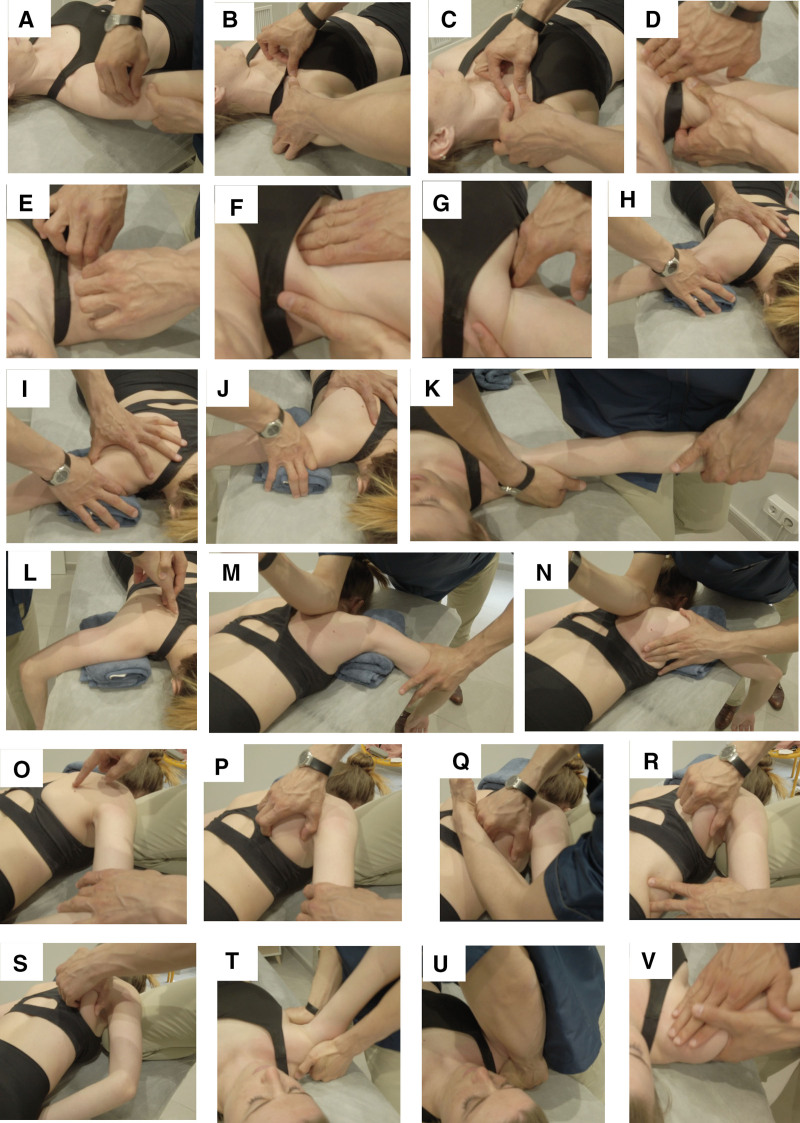
Manual therapy protocol for GIRD treatment. Digital superficial glides on the superficial deltoid fascia (A). Anteroposterior (B) and craniocaudal (C) mobilizations of the clavicle from sternocostoclavicular to acromioclavicular joints. Myofascial release using direct manual pressure of subclavian muscle (D), pectoralis muscle (E), pectoralis minor muscle (F), and subscapularis muscle (G). Myofascial releases of external rotators with glenohumeral decoaptation facilitating triangular space, quadrangular space and triceps hiatus (H–J). Myofascial releases external rotators with glenohumeral decoaptation (K). Myofascial releases of the scapular musculature: rhomboids, trapezius (L–N), subscapularis including mobilization with a scapulothoracic joints decoaptation, and angular of the scapula (O–S). The treatment finished with a technique of supine mobilization of the posterior capsule in 90º shoulder flexion and adduction (T, U) and circumductions (V). GIRD = glenohumeral internal rotation deficit.

The patient photographed in Figure 1 gave her consent for publication since photographs preserve anonymity.

### 2.3. Sample

All patients with shoulder pain who attended the consultation, accepted, and agreed to participate in the study between October 1, 2020 and March 31, 2021 were included. The shoulder pain in the patients was understood as when they manifested an unpleasant sensory and emotional experience directly associated with actual or potential tissue damage^[20]^ in their painful shoulder and imaging and image evidence to support it, following the criteria of the International Statistical Classification of Diseases and Related Health Problems (ICD).^[21]^ Exclusion criteria consisted of being under 18 years of age, having shoulder pain in both shoulders and excluded all those patients with a joint prosthesis in at least one of the 2 shoulders. Tables 1 and 2 describe the composition of the evaluated patient group and their shoulder pain etiology, respectively.

**Table 1:** Anthropometric and sociodemographic description of the sample evaluated.

Anthropometric data	Mean ± SD (min–max)	
Age (years)	52.8 ± 12.8 (25–75)	
Size (cm)	168.9 ± 8.5 (156–189)	
Weight (kg)	68.8 ± 14.1 (44–102)	
BMI (kg/m^2^)	24 ± 3.8 (17.6–33.1)	
Sociodemographic data		n
Sex	Male	18
	Female	17
Marital status	Single	9
	Married	24
	Separated	1
	Widower	1
Dominance	Right-handed	31
	Left-handed	4
Painful shoulder	Right	23
	Left	12
Coincidence between the painful shoulder and the dominance	Yes	20
	No	15
Evolution	Up to 6 week	8
	Up to 12 weeks	8
	Up to 6 months	6
	More than 6 months	13
Previous surgeries	Yes	1
	No	34

BMI = body mass index.

**Table 2:** Shoulder pain etiology based on the radiological findings.

Type of injury	Radiological findings	n	%*
Acromioclavicular joint-related injuries	Abnormal joint	12	34.29
Biceps-related injuries	Tenosynovial fluid in the sheath	10	28.57
	Labrum tear	12	34.29
	Tendinopathy	9	25.71
	Abnormal tendon location	4	11.43
Bone-related injuries	Bankart	9	25.71
	Osteophytes on the humerus	5	14.29
	Hill Sachs	7	20.00
	Fracture and/or other post-traumatic/congenital deformity of the humerus	2	5.71
	Osteophytes on the glenoid fossa	1	2.86
	Bennett lesion	1	2.86
	Fracture and/or other post-traumatic/congenital deformity of the glenoid fossa	1	2.86
Bursa-related injuries	Fluid in subacromial-subdeltoid bursa	20	57.14
	Fluid in subcoracoid bursa	9	25.71
Cartilage-related injuries	Glenoid cartilage defect	2	5.71
	Humerus cartilage defect	1	2.86
Glenohumeral joint	Effusion	11	31.43
	Synovitis	4	11.43
	Specific signs of capsulitis	2	5.71
Rotator cuff-related injuries	Tendonopathy	31	88.57
	Partial rupture	11	31.43
	Complete rupture	5	14.29
	Calcifications	7	20.00

* The percentages denote the number of patients that showed each radiological finding respect to the 35 patients included in this study.

For the sample size calculation, the GRANMO calculator was used (version 7.12, Municipal Institute for Medical Research, Barcelona, Spain). The minimum sample size was calculated to be 35 patients for detecting at least a difference between IR_1_ and IR_0_ of 4º, assuming an alfa risk of 0.05, considering that it is the probability of rejecting the null hypothesis when it is true, and a beta risk of 0.2, taking into account that it is the probability of accepting the null hypothesis when it is false, with a standard deviation (SD) of 7º and a dropout rate of 30%. Thus, a total of 35 Caucasian patients (Tables 1 and 2) agreed to be treated in the first consultation.

### 2.4. Measurements

All participants underwent a physical examination by physiotherapist with one more than 20 years of experience (RJ-L), followed by anthropometric and sociodemographic data that is sex, age, height, weight, occupation, marital status, descent, previous surgeries, and sport habits (documented via questionnaire and by means of a structured interview). The IR of each shoulder was measured in supine position on a physiotherapy bench. The shoulder was held by the examiner at 90° abduction with 90° flexion in the elbow. Then, an assistant placed a clinical Absolute + Axis^TM^ goniometer (Baseline Evaluation Instruments, Irvington, NY) in the mid-point of the distal end of the vertically held forearm (neutral position). The examiner internally rotated the glenohumeral joint while stabilizing the scapula on the bench to avoid compensatory movements. When the scapula began to move into protraction or anterior tilt, the measurement endpoint was considered to have been reached, and the respective IR value was noted (pretreatment IR or IR_0_). The order of shoulders to be assessed was randomized. The assessment of IR was performed by the same person (RJ-L). Afterwards, each patient underwent orthopedic examination by an orthopedic surgeon with 17 years of experience (JT-G). Jobe test, Gerber test, Patte test, lift-off test (Lag sign), cross shoulder adduction test, Yergason test, anterior apprehension test and the presence of scapular dyskinesia were performed following the methodology previously described.^[22,23]^

### 2.5. Treatment

**Figure 2.**
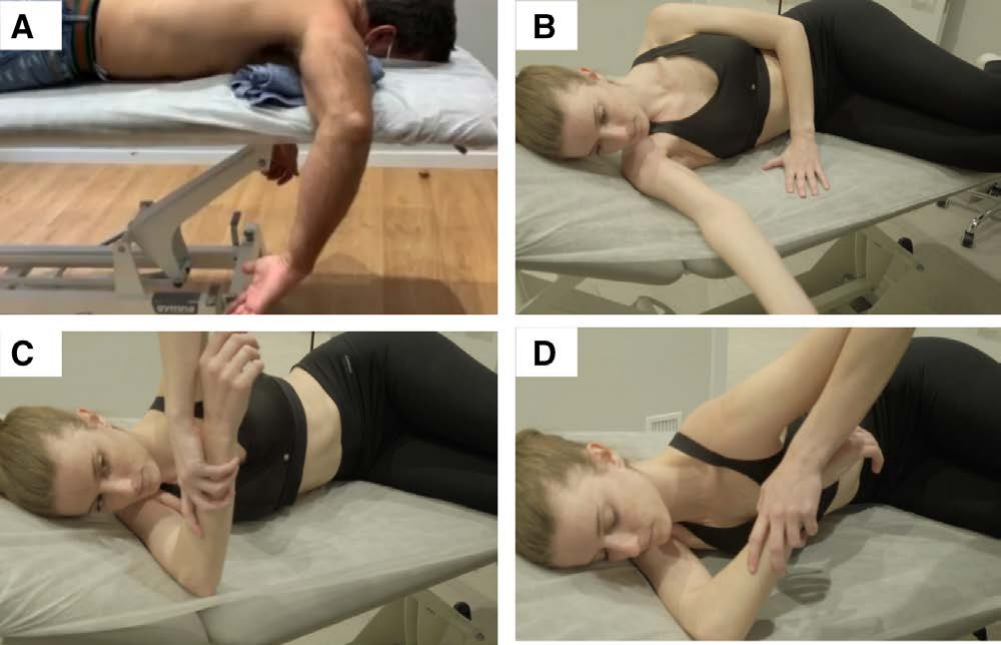
Exercises to be performed by treated patients. Gold standard therapeutic subscapular exercise (A). It is important to note that a towel should be used for this exercise. The sleeper stretch: shoulder 90-degrees abduction and shoulder retropulsion with scapular adduction (B), elbow 90-degrees flexion without losing shoulder 90-degrees abduction (C), and passive internal glenohumeral rotation is performed until the point of tension, gradually gaining joint balance for 2 minutes at a time (D). It is essential that a middle position between lateral decubitus homolateral to the side be treated and prone decubitus is achieved from the pure lateral decubitus position.

The treatment here is applied to the patients and is based on a manual therapy aimed at increasing the IR of a painful shoulder and reducing, at the same time, the GIRD. This kind of treatment has traditionally been used to improve the range of motion in situations of mobility deficits of the shoulder joint complex, such as GIRD.^[24–26]^ A treatment sequence including joint manual therapy techniques and soft tissue release techniques was proposed as a treatment in this study. In the supine position, first, the therapist performed gliding manoeuvers over the superficial deltoid fascia (Fig. 1A). This technique prepares the shoulder for the application of deeper maneuvers. Second, anteroposterior mobilization of the clavicle was carried out to mobilize the sterno-costo-clavicular joint and the acromioclavicular joint, preparing the shoulder joint complex for global mobilizations and facilitating the mobilization of the pectoral fascia (Fig. 1B and C). Third, the application of myofascial release of the shoulder joint complex techniques using direct manual pressure was done. This facilitates mobilization and the movement of these structures. The muscles were handled in the following order: subclavian muscle, pectoralis muscle, pectoralis minor muscle, and subscapularis muscle (Fig. 1D–G). Fourth, myofascial of the external rotators with glenohumeral decapitation was carried out in a prone position releasing the triangular space, quadrangular space, and triceps hiatus (Fig. 1H–J). Fifth, anterior and posterior, superior, and inferior glenohumeral inferior glide mobilization in abduction was performed in a prone position to facilitate glenohumeral recentralization (Fig. 1K). Sixth, scapular musculature myofascial releases (rhomboids, trapezius, subscapularis) (Fig. 1L–N) including mobilization with a scapulothoracic joints decoaptation, and the angle of the scapula (Fig. 1O–S). It is important to place a towel between the front side of the patient and the stretcher to avoid anteriorization of the humeral head. Seventh, the was terminated with a technique of supine mobilization of the posterior capsule in 90º shoulder flexion and adduction (Fig. 1T and U), with a good counteraction at the scapular level to avoid compensations, and circumductions (Fig. 1V). Immediately after treatment, IR of the painful shoulder was again measured as previously described (posttreatment IR or IR1). The IR was considered the outcome measure since it has demonstrated good reliability^[27,28]^ and validity^[28]^ in previous studies related to shoulder pathologies. The GIRD was calculated as the difference between the IR of the non-painful shoulder and the IR of the painful shoulder before (GIRD_0_) and after treatment (GIRD_1_). Finally, the patient was instructed to adapt the daily active biological stimulus at home to maximize the benefits of the proprioceptive and diagnostic manual therapy carried out in the consultation room. Thus, we exercised the subscapularis in the prone position, with the shoulder in 90º abduction to the detriment of the pectoralis major, ensuring the activation of the subscapularis and sliding of the posterior humeral head if we objectively control the anteriorization of the shoulder, placing a towel between the humeral head and the stretcher (Fig. 2A). We concluded by performing the sleeper stretch in an optimal and impeccable manner, the latter in front of the mirror to achieve an optimal visual impact (Fig. 2B–D).

### 2.6. The minimal clinically important difference calculation

The minimal clinically important difference (MCID) was estimated from a distribution-based calculation using Cohen *d*. The MCID calculation is based on the SD of the difference in the IR_1_ and the IR_0_. Following Cohen criteria,^[29]^ a MCID can be considered as clinically meaningful for any treatment difference ≥ 0.2 times the IR_0_ SD, with effect sizes of 0.2, 0.5, and 0.8 SD suggested as small, moderate, and large effects, respectively.

### 2.7. Statistical analysis

Data distribution was evaluated using Kolmogorov–Smirnov statistics with Lilliefors correction. Descriptive statistics are cited as means ± SD in case of normal distribution and as median and interquartile range in case of non-normal distribution for each of the variables that were calculated. A Student *t*-test considering the equality of variances was used to compare 2 groups and ANOVA was used to compare more than 2 groups. Pearson correlation to determine potential relationships between patients’ age, height, weight, and body mass index with GIRD were calculated. A paired Student *t*-test was used to compare the GIRD of each patient before and after the treatment. All analyses were performed using STATA statistical software, release 11 (StataCorp. 2009, StataCorp LP, College Station, TX). A significance level a priori was set at α = 0.05.

## 3. Results

**Figure 3.**
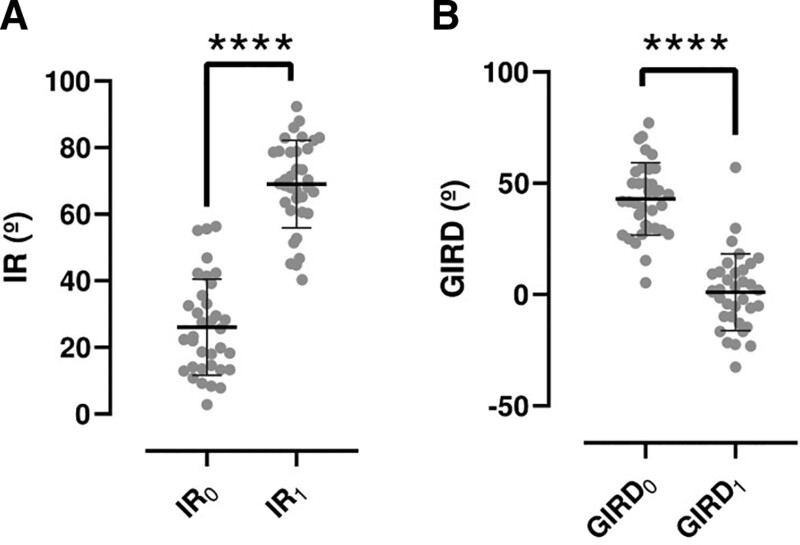
(A) Internal rotation (IR) in patients with shoulder pain before (IR_0_) and after treatment (IR_1_). (B) Glenohumeral internal rotation deficit (GIRD) in patients with shoulder pain before (GIRD_0_) and after treatment (GIRD_1_). The bars represent mean ± standard deviation. ****: *P*-value < .0001.

Treatment of the patients significantly increased the IR of the painful shoulder in all the patients (*P*-value < .0001) (Fig. 3A). So, the mean IR_0_ was 26.09 ± 14.46º (23.64–28.53), and after the treatment the mean IR_1_ was 67.98 ± 15.03º (65.48–70.52). Cohen *d* for IR_1_ and IR_0_ was 0.172. The mean difference after the treatment (IR_1_–IR_0_) was 41.89 ± 14.74º (39.4–44.39). The MCID (as 0.5 × IR_0_ SD) was 7.23º.

GIRD before and after treatment and improvement percentage are represented in Figure 3B. As can be seen, the treatment significantly reduced GIRD (*P*-value < .0001) (Fig. 3B). So, the mean GIRD_0_ was 42.95 ± 16.26º (40.2–45.7), and after the treatment the mean GIRD_1_ was –1.05 ± 17.18º (-3.96-1.85). Cohen *d* for GIRD_1_ and GIRD_0_ was 2.63. The MCID (as 0.5 × GIRD_0_ SD) was 8.13º.

## 4. Discussion

In this study, we aim to describe the efficacy of the GIRD treatment in nonathlete population. Although the definition of GIRD an exact value is still controversial,^[6]^ a 20° side-to-side difference is commonly considered diagnostic of GIRD for athletes.^[7]^ According to our open clinical trial, 97.14% of patients with shoulder pain showed a GIRD equal to or higher than 20º.

GIRD treatment involves targeting posterior capsular thickening and the posterior rotator cuff muscular adaptations in the form of “sleeper stretches” (arm at 90° shoulder flexion) and “cross-body stretches” (no scapular stabilization). There are two kinds of treatment for GIRD depending on who performs the treatment. One of them can be autonomously performed by the patient and the other one is assisted by the clinician. The autonomous stretches represent a huge preventive^[30]^ and treatment^[17]^ tool for athletes with GIRD, however, they require a great deal of commitment on the part of the patients who are sports professionals committed to their sporting careers. That commitment is not to be taken for granted in non-sporting professional patients with shoulder pain, so a treatment assisted by the clinician represents the best choice.

The treatment was effectively able to increase the IR of the painful shoulder from the first consultation. The efficacy of the treatment is supported by the MCID, as the mean IR_1_ and its 95% confidence interval, 67.98 ± 15.03 (65.48–70.52)º, exceed the MCID (7.23º), then the intervention was notably effective.^[31]^ On the contrary, the treatment was significantly able to reduce the GIRD in the patients. Thus, this was again endorsed by the MCID since the mean GIRD_1_ and its 95% CI, –1.05 (–3.96º to 1.85º), included the zero line and was much lower than the MCID (8.13º), what means the intervention was a definitely negative effect of it.^[31]^ These results are in line with a recent meta-analysis that asserts that a conservative therapy based on stretch, passive joint and muscular mobilizations can improve the IR of the shoulder in overhead athletes with GIRD.^[32]^ It should be noted that our trial showed a compliance rate of 100%, no dropouts and slight discomfort when touching painful places as possible adverse effects. The treatment here used showed some advantages and disadvantages. Among the advantages, the manual therapy is a low-cost, fast and very effective approach.^[33,34]^ As a disadvantage, this treatment requires to be administered by a physiotherapist experienced in manual therapy and arthrokinematic biomechanical reasoning.

There are some limitations in our study. First, the number of patients treated is relatively small. Second, the ER of each shoulder was not considered. The sum of IR and ER gives rise to the total range of motion. Recently, Rose and Noonan^[7]^ defined a pathological GIRD, based on a conservative estimation, as an IR deficit of >20º with a loss in total range of motion of >5º when compared to the non-painful shoulder. This concept was not considered in this study since ER was not taken. Third, the IR was not measured overtime beyond the first consultation day when the painful shoulder was treated. This did not allow sufficient time to evaluate the evolution of the patients GIRD after the treatment or whether the patients showed good adherence to maintenance treatment.

In conclusion, the treatment administrated in this study significantly increased the IR of the treated and painful shoulder and reduced the GIRD from the first consultation. Further studies should be addressed to corroborate the results here described through randomized clinical trials comparing different types of treatments and considering only the nonathletic population.

## Acknowledgments

We wish to acknowledge Dr John Jairo Aguilera-Correa for his writing assistance, and Dr Amr A. Abdelkader from the International Centre for Orthopaedics and Neurosciences (Doha, Qatar) for his help in reviewing the manuscripts for language-related aspects, and Mr. José Luis Lara-Cabrero from Clínica CEMTRO (Madrid, Spain) for his help in interpreting the treatment sequence of specialized manual therapy.

## Author contributions

**Conceptualization:** Rafael Jácome-López, Javier Tejada-Gallego, José María Silberberg, Francisco García-Muro San José.

**Data curation:** Rafael Jácome-López, Javier Tejada-Gallego.

**Formal analysis:** Rafael Jácome-López, Javier Tejada-Gallego.

**Funding acquisition:** Rafael Jácome-López, Javier Tejada-Gallego.

**Investigation:** Rafael Jácome-López, Javier Tejada-Gallego, José María Silberberg, Fernando García-Sanz.

**Methodology:** Rafael Jácome-López, Javier Tejada-Gallego, José María Silberberg, Fernando García-Sanz.

**Project administration:** Francisco García-Muro San José.

**Resources:** Javier Tejada-Gallego.

**Software:** Rafael Jácome-López, Javier Tejada-Gallego.

**Supervision:** José María Silberberg, Francisco García-Muro San José.

**Validation:** Rafael Jácome-López, Javier Tejada-Gallego, Fernando García-Sanz, Francisco García-Muro San José.

**Visualization:** Rafael Jácome-López, Javier Tejada-Gallego, José María Silberberg, Fernando García-Sanz, Francisco García-Muro San José.

**Writing – original draft:** Rafael Jácome-López, Javier Tejada-Gallego, José María Silberberg, Fernando García-Sanz, Francisco García-Muro San José.

**Writing – review & editing:** Rafael Jácome-López, Javier Tejada-Gallego, José María Silberberg, Fernando García-Sanz, Francisco García-Muro San José.
